# The Fruit Proteome Response to the Ripening Stages in Three Tomato Genotypes

**DOI:** 10.3390/plants11040553

**Published:** 2022-02-19

**Authors:** Hyo-Gil Choi, Dong-Young Park, Nam-Jun Kang

**Affiliations:** 1 Department of Horticulture, Kongju National University, Yesan 32439, Korea; hg1208@kongju.ac.kr; 2 Department of Horticulture, Gyeongsang National University, Jinju 52828, Korea; teastory@korea.com; 3 Institute of Agriculture & Life Science, Gyeongsang National University, Jinju 52828, Korea

**Keywords:** 2-DE mass spectrometry, cellular transport, metabolism, protein synthesis, ribosome biogenesis

## Abstract

The tomato is a horticultural crop that appears in various colors as it ripens. Differences in the proteome expression abundance of a tomato depend on its genotype and ripening stage. Thus, this study aimed to confirm the differences in changes in the proteome according to four ripening stages (green, breaker, turning, and mature) of three tomato genotypes, i.e., yellow, black, and red tomatoes, using a gel-based proteomic technique. The number of protein spots shown as two-dimensional electrophoresis (2-DE) gels differed according to tomato genotype and ripening stage. A total of 286 variant proteins were determined using matrix-assisted laser desorption-time of flight (MALDI-TOF) mass spectrometry (MS) analysis, confirming 233 identified protein functions. In three tomato genotypes in each ripening stage, grouping according to the Munich Information Center for Protein Sequences (MIPS) functional categories confirmed the variant proteins involved in the following: energy processes (21%); metabolism (20%); protein fate (15%); protein synthesis (10%); a protein with a binding function or cofactor requirement (8%); cell rescue, defense, and virulence (8%); cellular transport, transport facilitation, and transport routes (6%); the biogenesis of cellular components (5%); cell cycle and DNA processing (2%); others (5%). Among the identified protein spots in the function category, two proteins related to metabolism, four related to energy, four related to protein synthesis, and two related to interaction with the cellular environment showed significantly different changes according to the fruit color by the ripening stage. This study reveals the physiological changes in different types of tomatoes according to their ripening stage and provides information on the proteome for further improvement.

## 1. Introduction

The tomato is a vegetable of the species of *Solanum lycopersicum* and contains good nutrients and phytochemicals [1]. It is cultivated worldwide, and it is often grown in a greenhouse during the winter season in Korea. Tomatoes vary in size, shape, and color. As the tomato ripens, the color changes depending on the genotype, and, depending on the color, it has various antioxidant functions [2,3].

Red tomatoes are generally classified into six ripening stages according to their color image classification: green, breaker, turning, pink, light red, and red [4,5]. Tomatoes of all colors have a high chlorophyll content without other pigments at the early stage of maturation, so they are all green, but as they ripen, they show various colors depending on the type of phytochemicals. The commercially available tomatoes in Korea are mainly red, yellow, and black, and various studies show that tomato color is affected by lycopene, β-carotene, and lutein [1,5,6,7,8,9,10]. In yellow fruit, the content of lycopene is very low, but the content of lutein and β-carotene is high [1,6,9,10]. On the contrary, in red fruit, the lutein content is very low, but the lycopene content is significantly higher [1,6,9,10]. Black fruit is known to have a significantly higher β-carotene content than red fruit [1,8].

Tomatoes are widely used as a model plant for genome analysis among horticultural crops [11]. Recently, genome analysis and gene expression studies related to tomato development and maturity have been conducted [12,13,14,15]. Studies of genetic analysis according to the maturity of the tomato are mostly about ethylene signal transduction, cell wall metabolism, and carotenoid biosynthesis [16,17]. Fruit maturity is a complex physiological process that causes biochemical changes, such as the accumulation of beneficial compounds and abundant nutrients, that affect human nutrition [18,19]. Proteins related to fruit maturity have been identified through mass spectrometry, after the separation of total protein as two-dimensional electrophoresis (2-DE), and proteomic analysis is generally studied mainly using the seeds, flesh, and skin of the fruit [20,21]. The degree of maturity can be confirmed by analyzing the proteome expressed in the rind at the green and mature stages of the fruit; in grapes, the coloration increases as they ripen, and the activity of glutamine synthetase increases [22,23]. In addition, enzymes involved in glycolysis, such as UTP-glucose-1-phosphate uridyltransferase, triosephosphate isomerase, glyceraldehyde-3-phosphate dehydrogenase, and aldolase, are known to be involved in respiration and malate biosynthesis by supplying sugar during fruit ripening [24,25]. In the case of cherry tomatoes, it was found that the activity of S-adenosylmethionine (SAM) synthase, which is involved in the amino acid metabolism of the rind, was significantly reduced in the fully mature rind, and the heat shock protein, which plays an important role under stress, increased in the rind as the maturation period passed [26]. Ethylene is a hormone that plays an important role in fruit maturity. The activities of ethylene-related enzymes such as 1-aminocyclopropane-1-carboxylate oxidase, SAM synthetase, and β-cyanoalanine synthase are high in the early stage of fruit ripening but decrease as ripening progresses, while some enzymes such as endopolygalacturonase, xyloglucan, endotransglucosylase/hydrolase protein 32, pectinesterase inhibitor, and glycine-rich proteins that are involved in cell wall metabolism during fruit ripening have shown significant quantitative changes in fully mature fruit [18,27]. It has been reported that the activity of acid invertase increases as maturation progresses in tomatoes, the activities of SAM synthetases and phosphoglycerate dehydrogenase decrease constantly, and there is no difference in lipocalin [26,28]. 

As mentioned, through many studies [1,5,6,7,8,9,10], it is known that the color of tomatoes changes depending on the difference in the content of pigments such as lycopene, β-carotene, and lutein during the ripening process. However, studies on the difference in protein expression during the ripening stage of tomatoes based on fruit color are rare. Thus, this study was carried out to confirm the difference in metabolism and proteome abundance expression during the four ripening stages with three tomato genotypes, with yellow, black, and red tomatoes.

## 2. Materials and Methods

### 2.1. Plant Material

Tomato (*Solanum lycopersicum*) genotypes, i.e., the yellow tomato (Sugaryellow, Asia Seed Ltd., Seoul, Korea), the black tomato (Sinheuksu, Asia Seeds Ltd., Seoul, Korea), and the red tomato (Rapsodie, Syngenta Korea Seeds Ltd., Seoul, Korea), were used as the proteome analysis samples in this experiment. The three tomato genotypes grown under identical conditions in a greenhouse were harvested in triplicate repeats for each ripening stage: green (7–10 days after pollination, DAP), breaker (15–20 DAP), turning (25–30 DAP), and mature (35–40 DAP). At one harvest, a fruit sample of 1 kg was harvested for each genotype at each ripening stage. Analysis for total protein extraction and protein identification in these harvested samples was independently performed at each harvest.

### 2.2. Protein Extraction 

After completely grinding 20 g of tomato samples harvested at each ripening stage with liquid nitrogen, 50 mM ethylenediaminetetraacetic acid (EDTA), 0.7 M sucrose, 0.1 M KCl, 2% mercaptoethanol (*v*/*v*), and 15 mL of 0.5 M Tris-HCl buffer solution containing 1 mM phenylmethane-sulphonyl fluoride (PMSF) were added, and polyvinylpolypyrrolidone (PVPP) corresponding to 1% of the sample was added for protein extraction. The mixture was vortexed with an equal volume of tris-saturated phenol and was centrifuged at 28,000× *g* for 20 min at 4 °C (64R Centrifuge; Beckman Coulter Inc., Brea, CA, USA), and only the supernatant was separated. Ten milliliters of phenol were added to the extracted protein and suspended, and it was centrifuged to obtain a phenol layer. Methanol containing 0.1 M ammonium acetate was added in an amount 4 times the volume of the phenol layer to homogenize and precipitate it at −20 °C for 24 h. Ten milliliters of 80% acetone were added to the precipitate obtained by centrifugation at 28,000× *g* for 15 min at 4 °C. The process of obtaining a precipitate after the suspension was repeated 3 times. The extracted substance was stored at −20 °C by adding 1 mL of 80% acetone.

### 2.3. Protein Quantitation 

The extracted protein was quantified using a two-dimensional electrophoresis (2-DE) Quant kit (GE Healthcare, Piscataway, NJ, USA). The protein precipitated in 80% acetone was centrifuged to completely remove the acetone, dissolved in a rehydration solution [7 M urea, 2 M thiourea, 4% 3-[(3-Cholamidopropyl)dimethylammonio]-1-propanesulfonate hydrate (CHAPS), 2 M dithiothreitol (DTT), and 0.5% IPG buffer pH 4–7 (GE Health, Waukesha, WI, USA)], and centrifuged for 10,000× *g* for 2 min at 4 °C (64R Centrifuge; Beckman Coulter Inc., Brea, CA, USA). Five hundred microliters of co-precipitant (GE Healthcare, Piscataway, NJ, USA) were then added and mixed, and the mixture was kept at room temperature for 3 min. Five hundred microliters of co-precipitant (GE Healthcare, Piscataway, NJ, USA) were added once more, and the mixture was lightly suspended. The supernatant was completely removed by centrifugation (Allegra 64R, Beckman, Fullerton, CA, USA) at 10,000× *g* or more for 5 min. After adding 100 μL of a copper solution (GE Healthcare, Piscataway, NJ, USA) and 400 μL of distilled water to the supernatant, the mixture was mixed until the precipitate was completely dissolved. One milliliter of a working color reagent (GE Healthcare, Piscataway, NJ, USA) was added to each tube and immediately mixed. After remaining at room temperature for 20 min, the protein was quantified by measuring absorbance at 480 nm using a spectrophotometer (DU800, Beckman Coulter, Fullerton, CA, USA).

### 2.4. Two-Dimensional Electrophoresis (2-DE)

Each protein sample and rehydration solution [9 M urea, 2% CHAPS, 10 mM DTT, 0.5% IPG buffer, and 0.01% bromphenol blue (BPB)] were added to a 17 cm IPG gel strip (BioRad, Hercules, CA, USA) together. After loading this onto an 18 cm multi-focusing tray (BioRad, Hercules, CA, USA), 3 mL of mineral oil was overlaid and then rehydrated at 20 °C and 50 V conditions. For isoelectric focusing (IEF), the voltage was increased from 250 to 10,000 V for 3 h using a Protein IEF Cell (Bio-Rad, Hercules, CA, USA), and a focusing of the protein sample was performed until the voltage was finally changed from 10,000 to 60,000 V. After IEF, the strips were reacted for 20 min by adding 1% DTT to an equilibration buffer solution [50 mM Tris-HCl (pH 8.8), 6 M urea, 2% SDS, 20% glycerol, and 0.01% BPB], and the solution was then removed. Once again, the strips were reacted with an equilibrating buffer solution for 20 min with 2.5% iodoacetamide. The strips that had completed the equilibration operation were electrophoresed on a 12% SDS-PAGE gel with a Protein II Ⅺ electrophoresis device (Bio-Rad, Hercules, CA, USA) at 15 mA for 24 h. After electrophoresis, the gel was stained with coomassie brilliant blue (CBB) G-250. First, the gel was fixed in a 30% ethanol and 2% phosphoric acid (*v*/*v*) solution for 30 min, and this was repeated 2 times. The washing process was performed 3 times with an interval of 20 min with a 2% phosphoric acid (*v*/*v*) solution. After reacting for 30 min in 18% ethanol, 2% phosphoric acid (*v*/*v*), and 15% ammonium sulfate (*v*/*v*), the equilibration process for staining was then performed in a solution containing 1% CBB G-250 (*v*/*v*) for 72 h. The dyed gel was washed repeatedly in distilled water to decolorize it, until an appropriate image was obtained, and the washed gel was fixed in a solution containing 5% acetic acid (*v*/*v*) for image analysis.

The image analysis was performed by scanning the gel with a calibrated densitometer (GS-800, Bio-Rad, Hercules, CA, USA), and PDQuest software (Version 8.01; Bio-Rad, Hercules, CA, USA) was used to quantitatively measure and compare the spots with changes in the 2-DE gel.

### 2.5. Peptide Extraction

After coring the protein spots on the gel, to decolorize the gel spots, they were reacted with distilled water for 10 min, and the gel spots were then sequentially reacted with a 50% acetonitrile solution and a 100% acetonitrile solution for 10 min, respectively. In the last step of decolorization, the gel spots were reacted with a 0.1 M ammonium bicarbonate/50% acetonitrile solution for 30 min. The decolorization process was repeated until the gel became transparent and then dried using a vacuum centrifuge (Scanspeed 32, Gyrozen, Daejeon, Korea). For reduction and alkylation of the dried gel spots, a 10 mM DTT and 1 M ammonium bicarbonate solution was added to the dried gel spot and reacted at 56 °C for 45 min. Afterward, a 55 mM iodoacetamide and 0.1 M ammonium bicarbonate solution was added and reacted for 30 min at room temperature in the dark. The reaction of the gel spot with the 0.1 M ammonium bicarbonate/50% acetonitrile solution for 30 min was repeated twice, and the gel spot was then dried again using a vacuum centrifuge. The digestion buffer containing trypsin (50 ng/μL trypsin/0.1% n-octyl-β-D-glucopyranoside/25 mM ammonium bicarbonate) was absorbed into the gel spot that had been reduced and alkylated, so that the trypsin solution would absorb into the gel. The trypsin-free digestion buffer (0.1% n-octyl-β-D-glucopyranoside/25 mM ammonium bicarbonate) was then soaked in the gel, sealed with a wrap, and reacted at 37 °C for 16 h. After that, 30 μL of a solution with acetonitrile, water, and 10% trifluoroacetic acid, an extraction solution, were mixed in a ratio of 66:33:0.1 and added to the digested gel spot. After this process was repeated twice, the extract was obtained by vacuum centrifugation (Scanspeed 32, Gyrozen, Daejeon, Korea), which was used for drying. Thereafter, each sample was dissolved by adding a solution with 50% acetonitrile, 49% water, and 0.1% trifluoroacetic acid and stored at −20 °C until analysis.

### 2.6. Protein Identification 

A 1 μL matrix solution (500 μL of acetonitrile, 490 μL of water, 10 μL of 10% trifluoroacetic acid) was added to 1 μL of extracted peptide and mixed with a pipet. Afterwards, this was then loaded onto a plate for matrix-assisted laser desorption ionization time-of-flight mass spectrometry (MALDI-TOF MS) analysis, dried in a dark place, and then analyzed. MS and MS/MS analysis were performed with an ABI 4800 plus TOF-TOF mass spectrometer (Applied Biosystems, Framingham, MA, USA) using a 200 Hz ND:YAG laser operating at 355 nm. The protein was identified using MS/MS spectra and ProteinPilot v.3.0 (Applied Biosystems, Framingham, MA, USA) with the National Center for Biotechnology Information (NCBI). The identified protein was searched for 50 ppm of peptide and fragment ion mass tolerance, and the identified protein was classified according to the standard of the GO-MIPS Funcat Conversion Table (http://geneontology.org/external2go/mips2go; accessed on 25 January 2022).

### 2.7. Statistical Analysis

The data of this experiment were analyzed using analysis of variance (ANOVA) with Duncan’s multiple range test using a significance level of *p* ≤ 0.05, and the effects of the genotype and the ripening stage were analyzed with a two-way ANOVA in SAS (SAS Institute Inc., Cary, NC, USA).

## 3. Results

### 3.1. 2-DE Gel Imagery and a Matched Set of Spots in Different Tomato Cultivars

**Figure 1:**
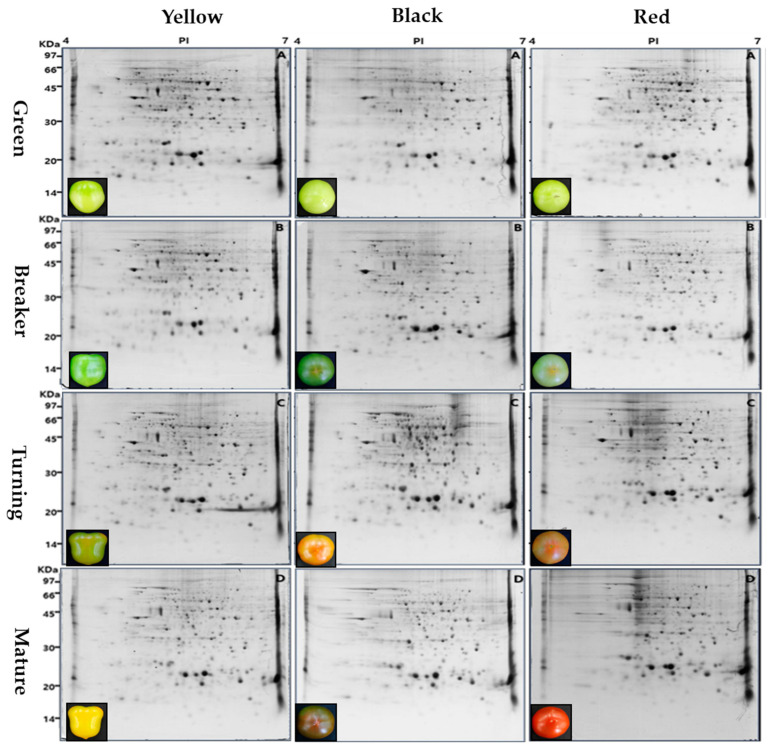
2-DE gel images of total proteins from fruits of the three tomato genotypes, i.e., yellow (Sugaryellow), black (Shinheuksu), and red (Rapsodie), in each ripening stage (green, breaker, turning, and mature). The molecular masses in kilodaltons (kDa) of pre-stained protein markers are shown on the left, and the linear range of isoelectric points (pI) is shown on the top.

The differences in protein pattern among the fruits of the three tomato genotypes during the ripening stages were confirmed using 2-DE gel images. According to the fruit color, the abundance of spots was different at each ripening stage. Compared to the yellow tomatoes, black and red tomatoes had a low number of protein spots at the turning and mature stages (Table 1 and Figure 1). 

**Figure 2:**
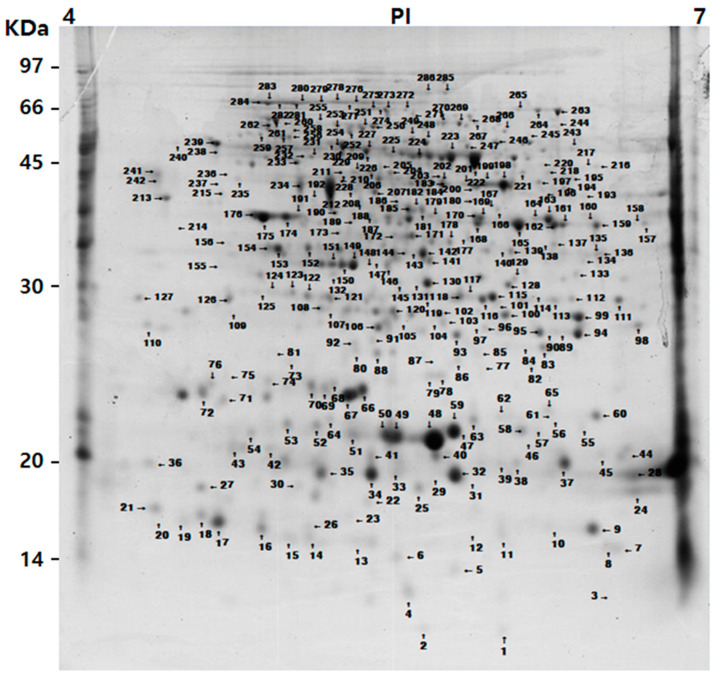
The gel was used to build the master gel. The molecular masses in kDa of pre-stained protein markers are shown on the left, and the linear range of pI is shown on the top.

The resulting protein spots can be identified through visual cross inspection of a new gel image with an already analyzed master gel [29]. Therefore, the identification of proteins of all color fruits was analyzed based on the master gel that used the gel of the mature stage of the yellow tomatoes with a high spot matching ratio (Figure 2). 

The peptides extracted through the gel digestion process by trypsin treatment of 286 spots capable of coring were identified using MALDI-TOF MS. A total of 286 protein spots were classified and identified in the NCBI database. Except for 12 spots, all of them (matching ratio: 96%) were identical to the proteins identified in the tomato. The remaining spots were analyzed as proteins extracted from cucumber, cacao, and corn (Table S1). Among the 274 identified protein spots, 223 protein spots had known functions. In the yellow tomatoes, 351, 308, 401, and 372 spots were detected in the green, breaker, turning, and yellow mature stages, respectively. In the same ripening stage as the yellow tomatoes, the spots of 256, 287, 333, and 311 were detected in the black tomatoes, and the spots of 398, 399, 334, and 257 were detected in the red tomatoes, respectively. The highest number of spots were detected in the turning stage of the yellow tomatoes. The highest number of matched spots was 364 in the mature stage of the yellow tomatoes, and the matching ratio was 97.8% higher. On the other hand, the number of matched spots in the black tomatoes was 201 in the turning stage, and the matching ratio was 64.6% lower. Compared with the yellow tomatoes with a high matching ratio of spots in the mature stage, the black and red tomatoes had a higher spot matching ratio in the early ripening stages (green and breaker stages) (Table 1). 

### 3.2. Classified and Identified Protein Spots

**Figure 3:**
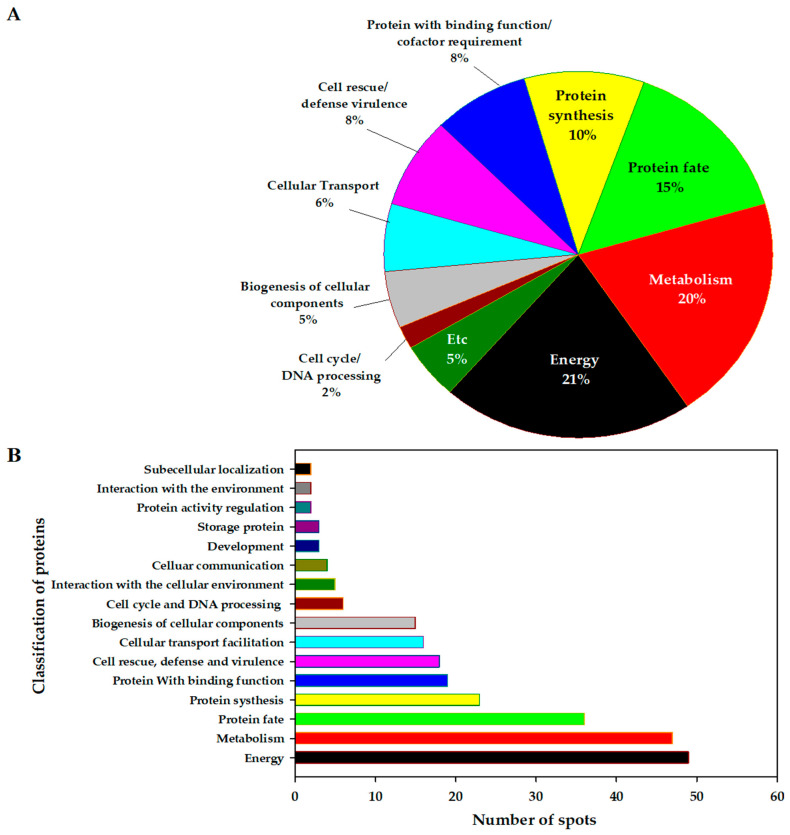
General information on differentially represented proteins according to the GO-MIPS fun cat conversion table. (**A**) Percentage of identified proteins and (**B**) assignment of the identified spots according to the functional category.

The majority of identified proteins spots were classified as related to energy (21%), metabolism (20%), protein fate (15%), protein synthesis (10%), a protein with a binding function/cofactor requirement (8%), cell rescue defense and virulence (8%), cellular transport (6%), cellular biogenesis (5%), cell cycle and DNA processing (2%), or other (5%) (Figure 3A). The energy-related proteins such as 3-phosphate dehydrogenase (Spots 157, 159, 162, 165, and 169), enolase (Spots 220, 222, 223, 224, and 225), phosphoglycerate kinase (Spots 193, 197, and 202), malate dehydrogenase (Spots 144, 161, 163, 165, and 169), alcohol dehydrogenase (Spot 114), and ATP synthase subunit β (Spots 226, 227, 228, 229, 230, 254, and 255) accounted for the largest percentage of the proteins whose functions were identified, and 49 spots were classified. Protein spots involved in metabolism were classified as methionine sulfoxide reductase A (Spots 82 and 83), cysteine synthase (Spot 140), glutamine synthase (Spots 187 and 189), S-adenosylmethionine synthase (Spots 200 and 204), and β-mannosidase (Spots 90, 125, 192, and 237). Thirty-five protein spots classified as related to protein fate were related to protein disulfide isomerase-like 2–1-like (Spots 186 and 188), calreticulin-3-like (Spots 218 and 219), chaperonin (Spot 252), proteasome subunit β-type (Spots 84 and 92), and proteasome subunit α-type (Spots 102, 103, 112, and 123 spots). Spots related to protein biosynthesis contained 40S ribosomal protein (Spots 8 and 75), 50S ribosomal protein (Spot 71), 60S ribosomal protein (Spots 27, 96, 272, and 275), and elongation factor Tu (Spots 194 and 195). Spots related to a protein with a binding function or cofactor requirement contained harpin binding protein (Spots 153 and 154), heme-binding protein (Spot 89), and ran binding protein (Spots 153 and 154). In addition, protein spots with functions related to cellular communication, development, protein activity regulation, and interaction with the environment were identified and classified (Table S1 and Figure 3B).

### 3.3. Differential Protein Expression Abundance According to Genotypes and Ripening Stages

Among the proteins whose functions have been identified, the proteins that show a distinct pattern of component change according to the tomato genotypes of yellow, black, and red during ripening stages were glutamine synthase (Spots 187 and 189), S-adenosylmethionine (SAM) synthase (Spots 200 and 204), glyceraldehyde 3-phosphate dehydrogenase (Spots 157, 159, 162, 168, and 264), phosphoglycerate kinase (Spots 193, 197, and 202), heat shock protein (HSP) 70 (Spots 279, 282, 283, and 284), and lipocalin (Spots 10, 61, and 65) (Table 2).

**Figure 4:**
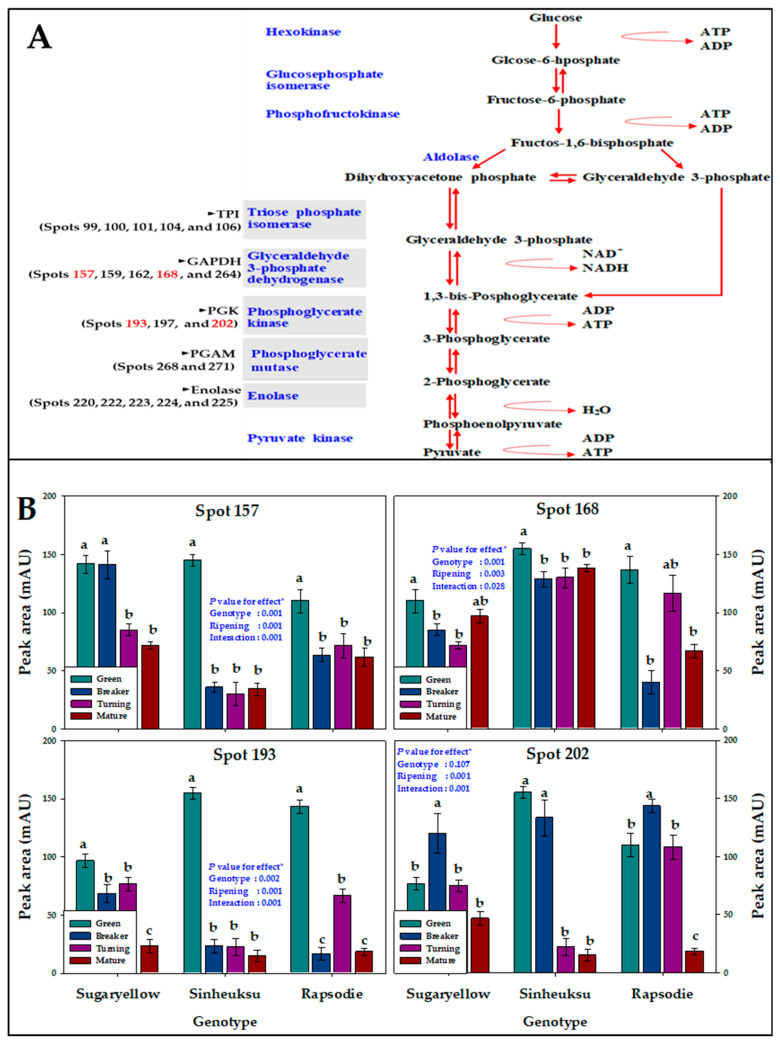
The proteome data for glycolysis and gluconeogenesis in fruits of the three tomato genotypes expressed in yellow (Sugaryellow), black (Shinheuksu), and red (Rapsodie) at the ripening stages. The expression changes were analyzed by one-way and two-way ANOVA, and vertical bars indicate standard deviation (*n* = 3). * *p* values were determined by two-way ANOVA. For each spot, the bars with different letters are significantly different according to Duncan’s multiple range test (*p* = 0.05). (**A**) Functional positions of identified spots in the glycolysis pathway. (**B**) Relative abundance of multiple spots related to GAPDH (Spots 157 and 168) and PGK (Spots 193 and 202) in the energy of the protein identification in Table 2.

A large number of protein spots were identified as having functions for enzymes involved in glycolysis and gluconeogenesis such as triosephosphate isomerase (TPI), glyceraldehyde 3-phosphate dehydrogenase (GAPDH), phosphoglycerate kinase (PGK), phosphoglycerate mutase (PGAM), and enolase (Figure 4A). Among the enzymes involved in glycolysis and gluconeogenesis, Spots 157, 168, 193, and 202, related to GAPDH and PGK enzymes, showed significant differences in expression abundance depending on the genotype and ripening stage (Figure 4B). In the case of the GAPDH-related Spot 157, the yellow genotype was highly expressed up to the breaker stage, whereas the black and red genotypes were rapidly reduced in expression abundance at that stage. Unlike Spot 157, another GAPDH-related spot (168) was relatively high in abundance until the mature stage in the black genotype compared with the other genotypes, and the yellow genotype did not drop sharply until the mature stage. On the other hand, in the red genotype, the expression abundance of Spot 158 showed a large change according to the ripening stages. The expression abundance of Spot 193 related to PGK was sharply decreased in the breaker stage in the black and red genotypes but did not drop rapidly until the turning stage in the yellow genotypes. On the other hand, the PGK-related Spot 202 was high in abundance in the red genotype until the turning stage, and the abundance in the black genotype was sharply decreased in the turning stage. Spot 202 of the yellow genotype was not expressed higher in the entire ripening stage compared with the other genotypes but did not drop sharply (Figure 4B). 

**Figure 5:**
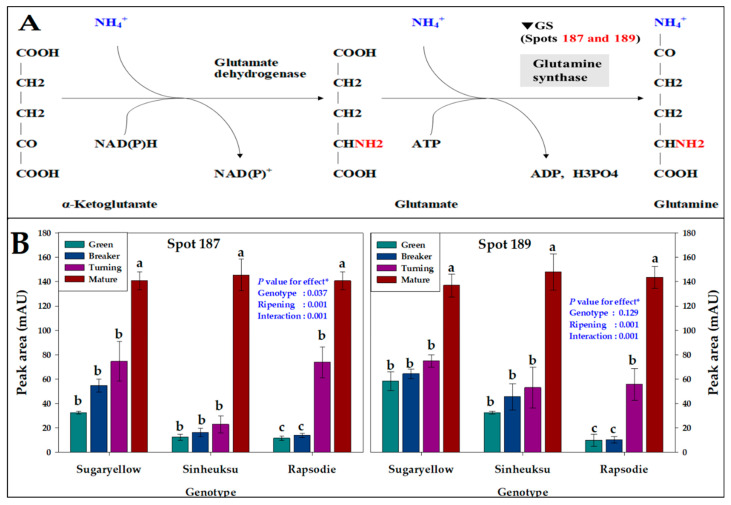
The proteome data for glutamine synthase of amino acid metabolism in the fruits of the three tomato genotypes, expressed in yellow (Sugaryellow), black (Shinheuksu), and red (Rapsodie) at the ripening stages. The expression changes were analyzed by one-way and two-way ANOVA, and vertical bars indicate standard deviation (*n* = 3). * *p* values were determined by two-way ANOVA. For each spot, the bars with different letters are significantly different according to Duncan’s multiple range test (*p* = 0.05). (**A**) Functional positions of identified spots in the glutamine synthase pathway. (**B**) Relative abundance of multiple spots related to glutamine synthase (GS) in the metabolism identification in Table 2.

The proteome data for the glutamine synthase of amino acid metabolism in the fruits of the three different tomato genotypes at the ripening stages are shown in Figure 5. Spots 187 and 189, which are related to the expression abundance of glutamine synthase (Figure 5A), an enzyme involved in glutamine metabolism, were significantly high at about 140 milli-absorbance unit (mAU) in all three varieties at the maturity stage. In addition, it was confirmed that the expression abundance of glutamine synthase increased as the ripening stage progressed for all three genotypes. On the other hand, in the early ripening stage, yellow fruits produced more glutamine synthase than black or red fruit genotypes. It was confirmed that the expression abundance of glutamine synthase was rapidly increased in the red tomatoes after the turning stage compared to the genotypes of other colors (Figure 5B).

**Figure 6:**
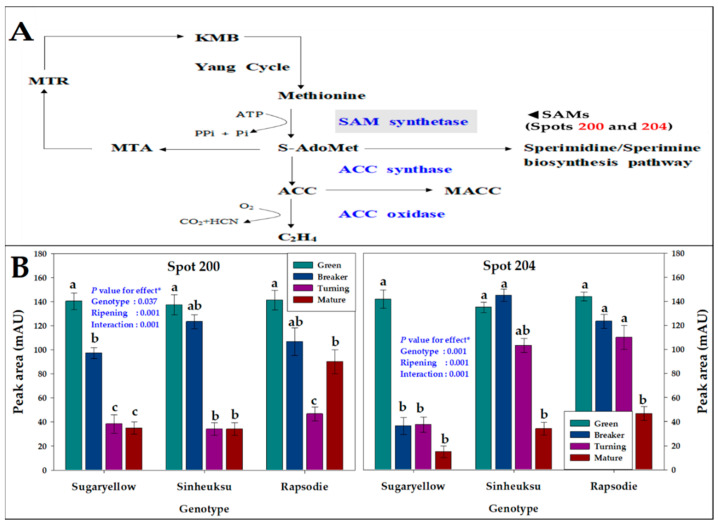
The proteome data for the spermidine/spermine pathway on ethylene biosynthesis in the fruits of the three tomato genotypes, expressed in yellow (Sugaryellow), black (Shinheuksu), and red (Rapsodie) at the ripening stages. The expression changes were analyzed by one-way and two-way ANOVA, and vertical bars indicate standard deviation (*n* = 3). * *p* values were determined by two-way ANOVA. For each spot, the bars with different letters are significantly different according to Duncan’s multiple range test (*p* = 0.05). (**A**) Functional positions of identified spots in the glutamine synthase pathway. (**B**) Relative abundance of multiple spots related to SAM synthetase in the metabolism identification in Table 2.

The proteome data for the spermidine/spermine pathway on ethylene biosynthesis in the fruits of the three different tomato genotypes at the ripening stages are shown in Figure 6. Spots 200 and 204 are related to SAM synthetase, an enzyme acting on methionine in ethylene biosynthesis (Figure 6A). These spots were highly active in the early ripening stage of the fruit, but their activity decreased as they ripened. Their expression abundance showed a large difference for each genotype in which a difference in fruit color appeared. In the yellow genotype, these spots decreased rapidly after the breaker stage, but the black and red genotypes were higher than the yellow genotype until the turning stage (Figure 6B).

**Figure 7:**
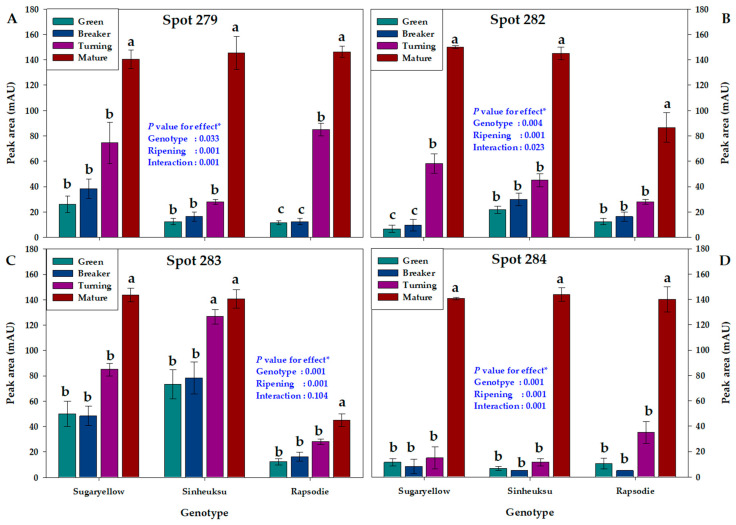
The proteome data for ribosome biogenesis in the fruits of the three tomato genotypes, expressed in yellow (Sugaryellow), black (Shinheuksu), and red (Rapsodie) at the ripening stages. The expression changes were analyzed by one-way and two-way ANOVA, and vertical bars indicate standard deviation (*n* = 3). * *p* values were determined by two-way ANOVA. For each spot, the bars with different letters are significantly different according to Duncan’s multiple range test (*p* = 0.05). (**A**) Heat shock protein 70 isoform 2 identified at Spot 279. (**B**) Heat shock protein 70 identified at Spot 282. (**C**) Heat shock protein 70 identified at Spot 283. (**D**) Heat shock protein 70 identified at Spot 284.

The three tomato genotypes with HSP-related protein spots, known as factors regulating ribosome biosynthesis, showed significant differences according to the ripening stage (Figure 7). The four HSP-related protein spots were highly expressed in the mature stage in all genotypes. Spot 279, identified as HSP 70 isoform 2, was high in all three genotypes in the mature stage, but low in the early ripening stage (green and breaker stages). In particular, the expression abundance of the red genotype in Spot 279 was very low, at about 10 mAU, until the breaker stage (Figure 7A). The HSP-related Spot 282 was expressed at a very low level, at ≤10 mAU, in the yellow genotype until the breaker stage and gradually began to increase at the turning stage. In the case of the black and red genotypes, Spot 282 showed no significant difference in expression until turning stage. The yellow and black genotypes increased to a very high level, to about 140 mAU, at the mature stage, whereas the red genotype increased to only about 80 mAU at this mature (Figure 7B). In the case of the HSP-related Spot 283, the red genotype was expressed by less than or equal to half of that of the other two genotypes over the entire ripening stage (Figure 7C). Among the protein spots related to HSP, Spot 284 showed the lowest expression until the breaker stage in all genotypes (Figure 7D). 

**Figure 8:**
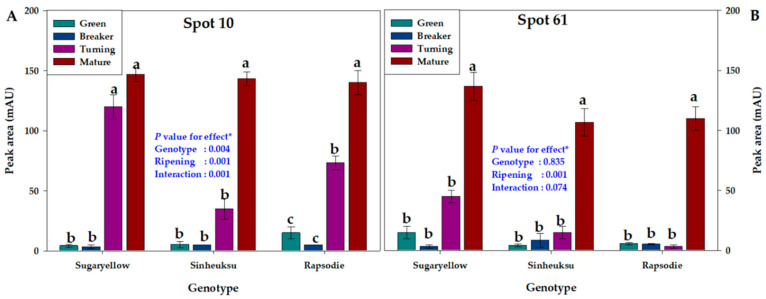
The proteome data for cellular sensing and response in the fruits of the three tomato genotypes, expressed in yellow (Sugaryellow), black (Shinheuksu), and red (Rapsodie) at the ripening stages. The expression changes were analyzed by one-way and two-way ANOVA, and vertical bars indicate standard deviation (*n* = 3). * *p* values were determined by two-way ANOVA. For each spot, the bars with different letters are significantly different according to Duncan’s multiple range test (*p* = 0.05). (**A**) Temperature-induced lipocalin identified at Spot 10. (**B**) Temperature-induced lipocalin identified at Spot 61.

Lipocalin is a protein that transports small hydrophobic molecules and is important for cell sensing and response. According to the tomato genotype and ripening stage, the lipocalin-related Spots 10 and 61 also showed different expression abundance patterns (Figure 8). These spots showed the highest expression in the mature stage. The yellow genotype showed a higher expression abundance in Spot 10 in the breaker stage than the black and red genotypes (Figure 8A). Spot 61 in the red tomatoes were hardly expressed (only ≤5 mAU) before the turning stage (Figure 8B).

## 4. Discussion

As tomatoes ripen, the color of the fruit changes from green to yellow, black, and red due to the actions of hormones and protein enzymes. Complex metabolic processes exist in these maturation processes [17,30,31]. In our experiment, which analyzed the proteome of three genotypes with different fruit colors at the ripening stage, 233 spots were identified and functionally classified (Table S1 and Figure 2). In the three tomato genotypes, the number of protein spots that changed significantly with each ripening stage was 9% of the total protein spots identified as 21 (Table 2 and Table S1). A previous study on cherry tomatoes also showed that only about 8% of the total spots on the master gel changed in strength during the fruit ripening stages [26]. In addition, it is reported that the differential expression abundance is about 10% as a result of transcriptomic analysis during the maturation of the tomato pericarp [32]. In the tomato, the central metabolic processes such as carbon metabolism, cell wall metabolites, pigments and flavonoids, and volatiles are regulated differently at different ripening stages [30]. In our experimental results, it was confirmed that the expression abundance of functional proteins in the tomatoes of different fruit genotypes was different at each ripening stage (Table 2). 

In all plants, glycolysis, which obtains energy by using self-synthesized glucose throughout the entire life cycle period, is a very important physiological function. It is reported that the activity of enzymes involved in glycolysis decreases as ripening progresses in fruits such as grapes (non-climacteric fruits) as well as tomatoes (climacteric fruits) [22,33]. In this study, it was also found that the expression abundance levels of protein spots related to enzymes such as GAPDH and PGK, which play a role in glycolysis and gluconeogenesis, decreased in the mature stage compared to the green stage (Figure 4). Protein spot 168, related to GAPDH, showed a relatively high abundance in black tomatoes in the entire ripening stage (Figure 4). The clear role of GAPDH in fruit maturation has not yet been elucidated [34]. However, in the results of our studies, it was confirmed that the activity of the protein related to the expression of GAPDH in the ripening stage of tomatoes was different depending on the color of the fruit. The GAPDH enzyme has been confirmed to be active in various stress conditions and regulate plant growth and development. It is known that the abundance of protein related to GAPDH and PGK in apples treated with ethylene decreased during the ripening stage [35]. Similar to our results from previous studies, protein spots related to GAPDH and PGK were decreased in ripe mango and apple fruits compared to unripe fruits, which indicated a slower carbon flow through glycolysis during fruit ripening [30]. 

The production of glutamine, an initial amino acid, from α-ketoglutarate produced in the tricarboxylic acid cycle occurring in mitochondria after glycolysis in the cytoplasm of plants is important for protein metabolism. We confirmed that the abundance of protein spots related to glutamine synthase, which plays an important role in glutamine production, increased rapidly during the mature stage in three tomato genotypes (Figure 5). In tomatoes, free amino acids increased rapidly during ripening, and the abundance of expression proteins changed significantly. L-glutamate enhances the umami taste of tomatoes as they ripen [36], and glutamate is reported as an important component of fruits by tomato variety [37]. According to our experimental results, the difference in fruit color affects the change in glutamine, which in turn will play a role in regulating the umami taste of the tomato. The *TDR4* gene is reported to affect the accumulation of certain amino acids, such as glutamate, during tomato ripening, reaffirming the importance of glutamate in tomato ripening [38].

Ethylene is a plant hormone that plays an important role in the ripening stage of tomatoes, which are climacteric fruits. Many studies have reported that the content of SAM synthetase in the biosynthetic pathway to ethylene is changed in the ripening of fruits [39,40,41]. The abundance of SAM synthetase-related protein spots was high in yellow fruits until the breaker stage, but the abundance of these protein spots in red fruit was continuously higher than it was in yellow fruits until the mature stage (Figure 6). In a tomato transformation experiment, it was reported that lycopene decreased when ethylene was reduced by regulating the ethylene-generating gene, whereas this decrease had no direct effect on carotenoids [42]. These results are similar to our study results.

All of the protein spots related to heat shock protein (HSP) showed a very high abundance at the mature stage rather than the early ripening stages, which seems to be closely related to the fully mature tomato. In particular, in the case of Protein Spot 283, the difference in abundance was significantly large according to the genotypes of different tomato colors (Figure 7). These results are considered to be related to the phytochemicals such as lycopene, the carotenoids, and the HSP of the fruit. HSP is reported to be important for the physiological response of fruits even after harvest, and it is reported to play a role in protecting the fruit from oxidative, high-temperature, chilling stress in particular [43,44,45]. 

Lipocalin is a ligand-binding protein that binds to small molecules as an important metabolite for the maturation of tomatoes because it regulates tolerance to oxidative and freezing stress [41]. It is reported that lipocalin plays an important role in regulating the growth and development of tomatoes and increases tolerance to abiotic stress [46]. Lipocalin-related protein spots (10 and 61) also showed a higher abundance in the late-ripening stage (mature) than in the early ripening stage (green). The abundance of lipocalin was higher in yellow tomatoes than in black and red tomatoes. In particular, the abundance of lipocalin was higher in yellow tomatoes than in red and black tomatoes in the breaker stage (Figure 8). We thought that, similar to HSP, lipocalin plays an important role in fully mature fruits, and its abundance is believed to be affected by the type of pigment that determines the fruit color. Carotenoids are involved in lipocalin synthesis [47,48], which supports differences in lipocalin abundance in the three tomato genotypes with different fruit colors.

## 5. Conclusions

The conclusions of the proteome analysis of the four ripening stages (green, breaker, turning, and mature) of tomato genotypes with yellow, black, and red fruit are as follows: 

First, during tomato ripening, intercellular metabolic reactions occur differently at each ripening stage, and it was shown that, among the functional classification of proteins, the number of spots, and abundance of energy and metabolism-related proteins was the highest. 

Second, our findings showed that the abundance of proteins involved in glycolysis (GAPDH and PGK) and ethylene (SAM synthetase) biosynthesis increased in the early ripening stages compared to the late ripening stage; it can be seen that the early ripening stage of fruit uses a high amount of energy, and that ethylene plays a significant role. The abundance of SAM synthetase involved in the ethylene biosynthesis of the yellow fruits decreased rapidly from the breaker stage, suggesting that the role of ethylene in the yellow fruits is decreased compared to other colored fruits from this period. 

Third, the abundance of the glutamine synthase, HSP, and lipocalin proteins was significantly increased in the late ripening stage compared to the early ripening stage. From this fact, we suggest that the umami taste of the fruit mainly increases at the mature stage, and it was shown in particular that the umami taste of yellow fruits is expressed faster than other colored fruits from the early stages, including the breaker and turning stages. We consider that the stress tolerance of yellow fruits is higher from the early ripening stage compared to other colored fruits because the abundance of abiotic stress-tolerance-related proteins in yellow fruits at early ripening is higher than in fruits of other colors. 

## Figures and Tables

**Table 1:** The match set of 2-DE in the fruits of three different tomato cultivars under four ripening stages.

Cultivar. (Fruit Color)	Ripening Stage ^z^	Spot ^y^	Matched Spot ^x^	Matching Ratio (%)
Sugaryellow (Yellow)	Green	351	269	76.6
	Breaker	308	242	75.6
	Turning	401	317	79.1
	Mature	372	364	97.8
Sinheuksu (Black)	Green	256	198	77.3
	Breaker	287	286	99.7
	Turning	333	211	63.7
	Mature	311	201	64.6
Rapsodie (Red)	Green	398	356	89.4
	Breaker	399	313	78.4
	Turning	334	244	73.1
	Mature	257	198	77.0

^z^ Fruits were harvested at the following four ripening stages: 7–10 days after pollination (DAP), defined as the green stage, 15–20 DAP, defined as the breaker stage (when the fruit starts to turn from green to orange), 25–30 DAP, defined as the turning stage (when the fruit starts to turn from orange to yellow or black or red), and 35–40 DAP, defined as the mature stage (when the fruit colors were yellow, black, or red). ^y^ The average number of spots in the gel of each sample (*n* = 6). ^x^ Spots omitting those with undefined shapes and areas based on the PDQuest (ver. 8.01) analysis.

**Table 2:** Differentially expressed proteins in tomatoes classified according to the MIPS functional category in the organism of *Solanum lycopersicum*.

Pot No. ^z^	Protein Identification ^y^	ID (NCBI)	Mr/pI	MP ^x^
Metabolism				
187	Glutamine synthase	Q42874	18,735/6.13	5
189	Glutamine synthase	Q42874	18,735/6.13	5
200	S-adenosylmethionine synthase 1	P43280	43,730/5.52	11
204	S-adenosylmethionine synthase 2	P43281	43,511/5.41	11
Energy				
157	Glyceraldehyde-3-phosphate dehydrogenase	O04891	32,097/5.93	10
159	Glyceraldehyde-3-phosphate dehydrogenase	O04891	32,097/5.93	16
162	Glyceraldehyde-3-phosphate dehydrogenase	O04891	32,097/5.93	16
168	Glyceraldehyde-3-phosphate dehydrogenase	gi|525314339	32,100/5.93	7
264	Glyceraldehyde-3-phosphate dehydrogenase	Q7Y0S2	28,957/5.70	8
193	Phosphoglycerate kinase	K4CHY3	42,263/5.78	9
197	Phosphoglycerate kinase, cytosolic-like	gi|460396822	39,627/6.49	8
202	Phosphoglycerate kinase	K4CHY4	50,592/7.66	13
Protein synthesis				
279	Heat shock protein 70 isoform 2	H1ZXA8	71,142/5.08	19
280	Heat shock protein 70 isoform 3	H1ZXA9	71,744/5.14	19
281	Heat shock protein 70 kDa protein 2-like	gi|460408280	71,141/5.08	18
282	Heat shock protein 70	A8W7B5	74,415/5.41	18
283	Heat shock protein 70	A8W7B5	74,415/5.41	16
284	Heat shock protein 70	A8W7B5	74,415/5.41	13
Interaction with the cellular environment				
10	Temperature-induced lipocalin	Q38JD4	21,509/6.16	10
61	Temperature-induced lipocalin	Q38JE1	21,303/5.96	5
65	Temperature-induced lipocalin	Q38JE1	21,303/5.96	7

^z^ Spot number as given on the 2-DE images in Figure 2 and identified as a functional protein in Table S1. ^y^ Protein identification on the function category was based on the ‘GO-MIPS funcat conversion table’ set up at the Munich Information Center for Protein Sequences (MIPS Institute). ^x^ Ratio of matched peptides.

## Data Availability

The data presented in this study are available in the article.

## Supplementary Materials

The following are available online at: https://www.mdpi.com/article/10.3390/plants11040553/s1, Table S1: Functional classification of proteins in three tomato genotypes at each ripening stage. Data were classified or unclassified according to the MIPS functional category in the organism.
