# Supplementary material for: The Fruit Proteome Response to the Ripening Stages in Three Tomato Genotypes
Source: Plants (Basel). 2022 Feb 19;11(4):553. doi: 10.3390/plants11040553 (PMC8877657; doi:10.3390/plants11040553)
Supplement: Supplementary file 1 [file plants-11-00553-s001.zip › plants-1592006-supplementary.pdf]

Table S1. Functional classification of proteins on three tomato color genotypes by ripening stages. Data were classified or unclassified according to the MIPS functional category in the organism.

Spot no[z]	Matched protein	ID (NCBI)	Organism	Mr/pI	MP[y]	SC[x]
**01 Metabolism 01.01 Amino acid metabolism**						
23	Glycine-rich protein	Q04130	*Solanum lycopersicum*	7327/9.92	4	60
81	Leucine-rich repeat extensin-like protein 3	gi|645235871	*Solanum lycopersicum*	40453/12.46	14	29
82	Methionine sulfoxide reductase A	G3JX11	*Solanum lycopersicum*	21346/6.09	4	25
83	Methionine sulfoxide reductase A	G3JX11	*Solanum lycopersicum*	21346/6.09	3	21
124	Leucine-rich repeat extensin-like protein 3	gi|645235871	*Prunus mume*	40453/12.46	13	27
139	Lactoylglutathione lyase-like	gi|460373807	*Solanum lycopersicum*	32955/5.95	7	17
140	Cysteine synthase	K4CVI4	*Solanum lycopersicum*	34335/5.93	14	49
145	Iactoylglutathione lyase-like	gi|460373807	*Solanum lycopersicum*	32955/5.95	13	36
148	lactoylglutathione lyase-like	gi|460373807	*Solanum lycopersicum*	32955/5.95	7	17
166	Aspartate-semialdehyde dehydrogenase-like	gi|460365718	*Solanum lycopersicum*	41785/7.01	3	10
177	Arginase 2	Arginase 2	*Solanum lycopersicum*	36943/5.60	13	38
178	Arginase 1	Arginase 1	*Solanum lycopersicum*	37140/5.96	9	21
180	Methylthioribose-1-phosphate isomerase	K4CJP1	*Solanum lycopersicum*	39543/5.66	9	25
187	Glutamine synthase	Q42874	*Solanum lycopersicum*	18735/6.13	5	25

Table S1. Continued.

Spot no[z]	Matched protein	ID (NCBI)	Organism	Mr/pI	MP[y]	SC[x]
**01.05 C-compound and carbohydrate metabolism**						
189	Glutamine synthase	Q42874	*Solanum lycopersicum*	18735/6.13	5	22
191	Cysteine synthase, chloroplastic/chromoplastic-like isoform 1	gi|460398434	*Solanum lycopersicum*	41261/5.41	9	32
198	Hop-interacting protein THI113	G8Z279	*Solanum lycopersicum*	37340/5.82	12	62
200	S-adenosylmethionine synthase 1	P43280	*Solanum lycopersicum*	43730/5.52	11	34
204	S-adenosylmethionine synthase 2	P43281	*Solanum lycopersicum*	43511/5.41	11	37
212	Hop-interacting protein THI111	G8Z278	*Solanum lycopersicum*	44905/5.10	8	18
249	N-carbamoyl-L-amino acid hydrolase-like	gi|460404363	*Solanum lycopersicum*	52976/5.81	9	17
13	Ribulose bisphosphate carboxylase small chain 3A/3C, chloroplastic	P07180	*Solanum lycopersicum*	20446/6.73	9	48
14	Ribulose bisphosphate carboxylase small chain 1, chloroplastic	P08706	*Solanum lycopersicum*	20522/7.57	8	31
90	Beta-mannosidase	Q8VWL8	*Solanum lycopersicum*	59536/8.76	8	10
125	Beta-mannosidase	Q8VWL8	*Solanum lycopersicum*	59536/8.76	13	14
141	Fructokinase-2	Q42896	*Solanum lycopersicum*	34969/5.76	9	27
142	Fructokinase-2	Q42896	*Solanum lycopersicum*	34969/5.76	16	55
158	UDP-glucuronic acid decarboxylase 1-like	gi|460382055	*Solanum lycopersicum*	38714/6.41	15	44

Table S1. Continued.

Spot no[z]	Matched protein	ID (NCBI)	Organism	Mr/pI	MP[y]	SC[x]
**01.07 Metabolism of vitamins, cofactors, and prosthetic groups**						
185	Coproporphyrinogen-III oxidase, chloroplastic-like	gi|460405900	*Solanum lycopersicum*	45252/5.92	8	15
**01.20 Secondary metabolism**						
95	Glutathione S-transferase-like	gi|460389800	*Solanum lycopersicum*	23708/5.98	6	45
98	Dehydroascorbate reductase	Q4VDN8	*Solanum lycopersicum*	23694/6.32	12	52
146	Aspartic proteinase A1-like isoform 2	gi|460395834	*Solanum lycopersicum*	55908/5.65	12	24
**02 Energy**	**02.01 Glycolysis and gluconeogenesis**					
99	Triosephosphate isomerase	K4BPK4	*Solanum lycopersicum*	27658/5.47	14	77
100	Triosephosphate isomerase	K4BPK4	*Solanum lycopersicum*	27658/5.47	10	35
101	Triosephosphate isomerase	K4BPK4	*Solanum lycopersicum*	27658/5.47	8	25
104	Triosephosphate isomerase	K4B3X5	*Solanum lycopersicum*	35043/6.45	17	59
106	Triosephosphate isomerase	K4B3X5	*Solanum lycopersicum*	35043/6.45	8	34
138	Fructose-bisphosphate aldolase	K4BAW0	*Solanum lycopersicum*	42656/8.15	7	20
157	Glyceraldehyde 3-phosphate dehydrogenase	O04891	*Solanum lycopersicum*	32097/5.93	10	30
159	Glyceraldehyde 3-phosphate dehydrogenase	O04891	*Solanum lycopersicum*	32097/5.93	16	48
162	Glyceraldehyde 3-phosphate dehydrogenase	O04891	*Solanum lycopersicum*	32097/5.93	16	53

Table S1. Continued.

Spot no[z]	Matched protein	ID (NCBI)	Organism	Mr/pI	MP[y]	SC[x]
168	Glyceraldehyde-3-phosphate dehydrogenase	gi|525314339	*Solanum lycopersicum*	32100/5.93	7	20
193	Phosphoglycerate kinase	K4CHY3	*Solanum lycopersicum*	42263/5.78	9	36
197	Phosphoglycerate kinase, cytosolic-like	gi|460396822	*Solanum lycopersicum*	39627/6.49	8	31
202	Phosphoglycerate kinase	K4CHY4	*Solanum lycopersicum*	50592/7.66	13	38
220	Enolase-like	gi|460408278	*Solanum lycopersicum*	48211/5.99	12	34
222	Enolase	P26300	*Solanum lycopersicum*	48054/5.68	13	40
223	Enolase	P26300	*Solanum lycopersicum*	48054/5.68	11	36
224	Enolase	P26300	*Solanum lycopersicum*	48054/5.68	11	36
225	Enolase	P26301	*Solanum lycopersicum*	48054/5.69	10	32
264	Glyceraldehyde 3-phosphate dehydrogenase	Q7Y0S2	*Solanum lycopersicum*	28957/5.70	8	18
268	2,3-bisphosphoglycerate-independent phosphoglycerate mutase-like	gi|460396104	*Solanum lycopersicum*	61278/5.59	13	21
271	2,3-bisphosphoglycerate-independent phosphoglycerate mutase-like	gi|460396104	*Solanum lycopersicum*	61278/5.59	11	19
**02.08 Pyruvate dehydrogenase complex**						
184	Pyruvate dehydrogenase E1 component subunit beta, mitochondrial-like	gi|460391698	*Solanum lycopersicum*	39804/6.18	7	21
245	Dihydrolipoyl dehydrogenase	K4DHT1	*Solanum lycopersicum*	53662/6.29	13	27

Table S1. Continued.

Spot no[z]	Matched protein	ID (NCBI)	Organism	Mr/pI	MP[y]	SC[x]
**02.10 Tricarboxylic-acid pathway**						
144	Malate dehydrogenase, chloroplastic-like isoform 2	gi|460380476	*Solanum lycopersicum*	43570/8.34	9	25
161	Malate dehydrogenase	K4CW40	*Solanum lycopersicum*	35703/5.91	15	62
163	Malate dehydrogenase	K4CGU8	*Solanum lycopersicum*	36287/8.73	11	38
165	Malate dehydrogenase	K4CW40	*Solanum lycopersicum*	35703/5.91	12	53
169	Malate dehydrogenase	K4CGU8	*Solanum lycopersicum*	36287/8.73	5	13
**02.13 Respiration**						
269	Succinate dehydrogenase [ubiquinone] flavoprotein subunit 1, mitochondrial isoform 2	gi|359476705	*Solanum lycopersicum*	69314/6.14	6	12
285	NADH dehydrogenase [ubiquinone] iron-sulfur protein 1, mitochondrial-like	gi|460409554	*Solanum lycopersicum*	80784/5.90	10	13
286	NADH dehydrogenase [ubiquinone] iron-sulfur protein 1, mitochondrial-like	gi|460409554	*Solanum lycopersicum*	80784/5.90	16	18
**02.16 Fermentation**						
114	Alcohol dehydrogenase homolog (Fragment)	Q40133	*Solanum lycopersicum*	26357/5.64	5	21
**02.30 Photosynthesis**						
67	Oxygen-evolving enhancer protein 2, chloroplastic	P29795	*Solanum lycopersicum*	27946/8.26	4	16
68	Oxygen-evolving enhancer protein 2, chloroplastic	K4CEP4	*Solanum lycopersicum*	27861/7.63	12	55
69	Oxygen-evolving enhancer protein 2, chloroplastic	P29795	*Solanum lycopersicum*	27946/8.26	3	10
70	Oxygen-evolving enhancer protein 2, chloroplastic	P29795	*Solanum lycopersicum*	27946/8.26	4	13

Table S1. Continued.

Spot no[z]	Matched protein	ID (NCBI)	Organism	Mr/pI	MP[y]	SC[x]
**02.45 Energy conversion and regeneration**						
74	Oxygen-evolving enhancer protein 2, chloroplastic	P29795	*Solanum lycopersicum*	27946/8.26	3	10
149	Oxygen-evolving enhancer protein 1, chloroplastic	P23322	*Solanum lycopersicum*	35154/5.91	12	52
150	Oxygen-evolving enhancer protein 1, chloroplastic	P23322	*Solanum lycopersicum*	35154/5.91	10	36
151	Oxygen-evolving enhancer protein 1, chloroplastic	P23322	*Solanum lycopersicum*	35154/5.91	5	20
97	Probable adenylate kinase 2, chloroplastic-like	gi|460392461	*Solanum lycopersicum*	32299/6.97	13	40
111	Probable ATP synthase 24 kDa subunit, mitochondrial-like	gi|460415839	*Solanum lycopersicum*	27754/8.69	9	42
226	ATP synthase subunit beta, chloroplastic	Q2MI93	*Solanum lycopersicum*	53491/5.28	24	60
227	ATP synthase subunit beta (Fragment)	A8Y6N6	*Solanum lycopersicum*	35930/5.18	7	23
228	ATP synthase subunit beta	K4BNR2	*Solanum lycopersicum*	59939/5.94	20	46
229	ATP synthase subunit beta	K4BX20	*Solanum lycopersicum*	59684/5.74	21	49
230	ATP synthase subunit beta	K4BX20	*Solanum lycopersicum*	59684/5.75	22	54
254	ATP synthase subunit alpha, chloroplastic	Q2MIB5	*Solanum lycopersicum*	55434/5.14	13	25
255	ATP synthase subunit alpha, chloroplastic	Q2MIB5	*Solanum lycopersicum*	55434/5.15	15	30
**04 Storage protein**	**04.01 Storage facilitating proteins**					
48	11S globulin subunit beta-like	gi|449480612	*Cucumis sativus*	54479/7.68	11	22

Table S1. Continued.

Spot no[z]	Matched protein	ID (NCBI)	Organism	Mr/pI	MP[y]	SC[x]
**10 Cell cycle and DNA processing 10.01 DNA processing**						
16	Histone H4	gi|195635409	*Zea mays*	20121/9.68	5	23
235	Putative DNA repair protein RAD23-3-like isoform 2	gi|460375404	*Solanum lycopersicum*	38831/4.67	7	26
236	Putative DNA repair protein RAD23-3-like isoform 2	gi|460375404	*Solanum lycopersicum*	38831/4.67	5	17
**10.03 Cell cycle**						
18	Profilin	Q8VWR0	*Solanum lycopersicum*	14187/4.71	5	37
19	Profilin-2	Q93YG7	*Solanum lycopersicum*	14362/4.63	4	24
**12 Protein synthesis 12.01 Ribosome biogenesis**						
8	40S ribosomal protein S21	K4AWT4	*Solanum lycopersicum*	9359/6.71	4	65
27	60S acidic ribosomal protein P2B-like isoform 2	gi|460411272	*Solanum lycopersicum*	11412/4.55	2	30
44	small ribosomal protein 4	gi|11228656	*Leucoloma serrulatum*	22211/10.53	11	52
71	50S ribosomal protein L12, chloroplastic-like	gi|460372985	*Solanum lycopersicum*	19579/6.11	4	20
75	40S ribosomal protein S7-like isoform 2	gi|460392051	*Solanum lycopersicum*	22114/9.80	8	32
77	Small heat shock protein, chloroplastic	Q95661	*Solanum lycopersicum*	26267/7.85	12	54
79	Small heat shock protein, chloroplastic	Q95661	*Solanum lycopersicum*	26267/7.85	3	11
96	60S ribosomal protein L10	Q3SC85	*Solanum lycopersicum*	25301/10.45	7	29

Table S1. Continued.

Spot no[z]	Matched protein	ID (NCBI)	Organism	Mr/pI	MP[y]	SC[x]
234	26S protease regulatory subunit 6A homolog	P54776	*Solanum lycopersicum*	47704/4.94	20	49
266	T-complex protein 1 subunit beta-like	gi|460411410	*Solanum lycopersicum*	57279/5.67	6	13
272	60S ribosomal protein L35a-3-like	gi|460388544	*Solanum lycopersicum*	13026/10.41	3	19
275	60S ribosomal protein L35a-3-like	gi|460388544	*Solanum lycopersicum*	13026/10.41	3	19
279	Heat shock protein 70 isoform 2	H1ZXA8	*Solanum lycopersicum*	71142/5.08	19	34
280	Heat shock protein 70 isoform 3	H1ZXA9	*Solanum lycopersicum*	71744/5.14	19	33
281	Heat shock cognate 70 kDa protein 2-like	gi|460408280	*Solanum lycopersicum*	71141/5.08	18	32
282	Heat shock protein 70	A8W7B5	*Solanum lycopersicum*	74415/5.41	18	26
283	Heat shock protein 70	A8W7B5	*Solanum lycopersicum*	74415/5.41	16	24
284	Heat shock protein 70	A8W7B5	*Solanum lycopersicum*	74415/5.41	13	18
**12.04 Translation**						
194	Elongation factor Tu, mitochondrial-like	gi|460404036	*Solanum lycopersicum*	49260/6.54	20	57
195	Elongation factor Tu	K4CUX6	*Solanum lycopersicum*	49257/6.54	19	54
**12.07 Translational control**						
58	Eukaryotic translation initiation factor 5A-2	Q9AXQ5	*Solanum lycopersicum*	17714/5.78	5	48
63	Eukaryotic translation initiation factor 5A-2	gi|350536449	*Solanum lycopersicum*	17717/5.78	3	14
64	Eukaryotic translation initiation factor 1A-like	gi|460368231	*Solanum lycopersicum*	16652/5.01	5	32

Table S1. Continued.

Spot no[z]	Matched protein	ID (NCBI)	Organism	Mr/pI	MP[y]	SC[x]
**14 Protein fate 14.01 Protein folding and stabilization**						
37	Class II small heat shock protein Le-HSP17.6	Q96489	*Solanum lycopersicum*	17670/6.32	9	67
38	Cytosolic class II small heat shock protein HCT2	O82545	*Solanum lycopersicum*	17379/6.75	2	16
39	Class II small heat shock protein Le-HSP17.6	Q96489	*Solanum lycopersicum*	17670/6.32	4	23
51	Class I small heat shock protein	G5DGD4	*Solanum lycopersicum*	17621/5.57	12	61
52	18.2 kDa class I heat shock protein-like isoform 1	gi|460401599	*Solanum lycopersicum*	17767/7.93	9	40
49	17.8 kDa class I heat shock protein	P30221	*Solanum lycopersicum*	17750/5.84	2	23
59	17.7 kD class I small heat shock protein	Q9SYU8	*Solanum lycopersicum*	17724/5.84	11	64
85	26.5 kDa heat shock protein, mitochondrial-like isoform 1	gi|460413910	*Solanum lycopersicum*	27273/8.90	4	17
186	Protein disulfide isomerase-like 2-1-like	gi|460368877	*Solanum lycopersicum*	39894/5.43	13	30
188	Protein disulfide isomerase-like 2-1-like	gi|460368877	*Solanum lycopersicum*	39894/5.43	11	24
196	Activator of 90 kDa heat shock protein ATPase homolog	gi|460407486	*Solanum lycopersicum*	39502/5.93	15	35
218	Calreticulin-3-like	gi|460387993	*Solanum lycopersicum*	51132/6.12	5	8
219	Calreticulin-3-like	gi|460387993	*Solanum lycopersicum*	51132/6.12	5	7
233	Hsp70-binding protein 1-like	gi|460401839	*Solanum lycopersicum*	43554/5.01	8	21
262	Protein disulfide-isomerase-like	gi|460389504	*Solanum lycopersicum*	55113/4.81	11	20
263	Heat shock protein STI-like	gi|460400417	*Solanum lycopersicum*	65415/5.99	26	45
265	Heat shock protein STI-like	gi|460400417	*Solanum lycopersicum*	65415/5.99	9	12

Table S1. Continued.

Spot no[z]	Matched protein	ID (NCBI)	Organism	Mr/pI	MP[y]	SC[z]
**14.07 Protein modification**						
1	Ubiquitin	A3FMH5	*Solanum lycopersicum*	8520/6.56	4	40
2	Hypothetical protein	gi|643727688	*Jatropha curcas*	17170/5.77	7	46
21	Hypothetical protein	gi|604332814	*Erythranthe guttata*	41053/6.59	12	23
122	Hypothetical protein	gi|565466538	*Capsella rubella*	49092/8.51	14	21
170	2-methylene-furan-3-one reductase	K4BW79	*Solanum lycopersicum*	40976/7.74	9	30
217	Hypothetical protein	gi|595853000	*Prunus persica*	126047/5.05	23	15
**14.10 Assembly of protein complexes**						
252	Chaperonin CPN60, mitochondrial-like	gi|460388438	*Solanum lycopersicum*	61436/5.80	18	31
**14.13 Protein degradation**						
84	Proteasome subunit beta type	K4CCD7	*Solanum lycopersicum*	25383/5.51	4	20
92	Proteasome subunit beta type	K4BP97	*Solanum lycopersicum*	23104/5.29	7	29
102	Proteasome subunit alpha type	K4BA40	*Solanum lycopersicum*	27369/5.63	7	38
103	Proteasome subunit alpha type	K4CXX8	*Solanum lycopersicum*	25659/5.39	7	39
112	Proteasome subunit alpha type	K4DC02	*Solanum lycopersicum*	27415/6.11	3	14
123	Proteasome subunit alpha type	K4B832	*Solanum lycopersicum*	26253/4.72	13	55
247	Probable mitochondrial-processing peptidase subunit beta-like	gi|565342500	*Solanum lycopersicum*	59352/5.96	17	32
248	Mitochondrial-processing peptidase subunit alpha-like	gi|460412524	*Solanum lycopersicum*	54873/6.05	13	32
258	26S protease regulatory subunit 6A homolog	P54776	*Solanum lycopersicum*	47704/4.94	22	51
259	26S protease regulatory subunit 6A homolog	P54776	*Solanum lycopersicum*	47704/4.95	7	17
267	Probable mitochondrial-processing peptidase subunit beta-like	gi|460386762	*Solanum lycopersicum*	59718/6.16	16	35

Table S1. Continued.

Spot no[z]	Matched protein	ID (NCBI)	Organism	Mr/pI	MP[y]	SC[z]
**16 Protein with binding function or cofactor requirement**		**16.01 Protein binding**				
107	Harpin binding protein 1	Q5QJB4	*Solanum lycopersicum*	30293/6.25	12	49
108	Harpin binding protein 1	Q5QJB4	*Solanum lycopersicum*	30293/6.25	7	27
135	Gamma carbonic anhydrase 1, mitochondrial-like	gi|460373230	*Solanum lycopersicum*	29418/6.25	7	30
**16.02 Peptide binding**						
153	Ran binding protein-1	Q94K24	*Solanum lycopersicum*	25229/4.88	6	40
154	Ran binding protein-1	Q94K24	*Solanum lycopersicum*	25229/4.88	9	54
**16.03 Nucleic acid binding**						
29	Glycine rich RNA binding protein 1a	L7Q568	*Solanum lycopersicum*	15927/5.53	10	76
30	Glycine-rich RNA-binding protein-like isoform 2	gi|460370204	*Solanum lycopersicum*	16495/5.58	7	44
78	Cold shock domain-containing protein 4-like isoform 5	gi|460370361	*Solanum lycopersicum*	17827/5.63	4	32
**16.13 C-compound binding**						
4	Uncharacterized protein	K4CMT6	*Solanum lycopersicum*	10091/5.40	2	24
**16.17 Metal binding**						
32	Beta myrcene/limonene synthase	G1JUH4	*Solanum lycopersicum*	69591/5.98	3	6
87	Sulfite oxidase	A5H1Q7	*Solanum lycopersicum*	43580/8.43	8	20
214	Ankyrin repeat domain-containing protein 2-like	gi|460369292	*Solanum lycopersicum*	37357/4.43	6	19
**16.19 Nucleotide binding**						
24	Fen-interacting protein 3	K4CGU0	*Solanum lycopersicum*	17280/6.74	4	17
273	CTR1-like protein kinase	Q5YKK6	*Solanum lycopersicum*	89563/5.66	12	14

Table S1. Continued.

Spot no[z]	Matched protein	ID (NCBI)	Organism	Mr/pI	MP[y]	SC[z]
**18 Protein activity regulation 18.02 Target of regulation**						
60	BTF3-like transcription factor	Q2PQI9	*Solanum lycopersicum*	17397/6.85	11	59
**20 Cellular transport, transport facilitation and transport routes 20.01Transported compounds**						
9	Nucleoside diphosphate kinase	P47921	*Solanum lycopersicum*	15726/7.04	6	35
12	Nucleoside diphosphate kinase	P47921	*Solanum lycopersicum*	15726/7.04	5	33
**20.03 Transport facilitation**						
40	ATP synthase epsilon chain, chloroplastic	Q2MI94	*Solanum lycopersicum*	14571/5.43	6	50
136	V-type proton ATPase subunit E-like	gi|460400045	*Solanum lycopersicum*	26667/6.33	10	29
137	V-type proton ATPase subunit E-like	gi|460400045	*Solanum lycopersicum*	26667/6.33	10	32
231	V-type proton ATPase subunit B2-like isoform 2	gi|460370437	*Solanum lycopersicum*	54298/4.99	8	14
232	V-type proton ATPase subunit B2-like isoform 2	gi|460370437	*Solanum lycopersicum*	54298/4.99	17	43
243	ATPase subunit 1 (mitochondrion)	gi|568245146	*Ajuga reptans*	55278/6.59	6	16
274	Vacuolar H+-ATPase A1 subunit isoform	Q84XW6	*Solanum lycopersicum*	68812/5.20	13	21
276	vacuolar H+-ATPase A1 subunit isoform	gi|350537129	*Solanum lycopersicum*	68817/5.20	12	19
277	Vacuolar H+-ATPase A1 subunit isoform	Q84XW6	*Solanum lycopersicum*	68812/5.20	14	27
278	Vacuolar H+-ATPase A1 subunit isoform	Q84XW6	*Solanum lycopersicum*	68812/5.20	13	24
**20.09 Transport routes**						
53	Tubulin-specific chaperone A-like	gi|460366584	*Solanum lycopersicum*	13096/4.92	7	48
244	Leucine aminopeptidase 2, chloroplastic	Q42876	*Solanum lycopersicum*	60082/8.18	15	29
246	Leucine aminopeptidase 2, chloroplastic	Q42876	*Solanum lycopersicum*	60082/8.18	11	20

Table S1. Continued.

Spot no[z]	Matched protein	ID (NCBI)	Organism	Mr/pI	MP[y]	SC[z]
**30 Cellular communication/signal transduction mechanism**		**30.01 Intracellular signalling**				
80	Guanine deaminase-like isoform 1	gi|460368541	*Solanum lycopersicum*	20639/5.27	6	36
**30.05 Transmembranesignaltransduction**						
36	Integrin-linked protein kinase family isoform 3	gi|590654217	*Theobroma cacao*	53850/9.28	14	22
**32 Cell rescue, defense, and virulence 32.01 Stress response**						
25	Superoxide dismutase [Cu-Zn], chloroplastic	P14831	*Solanum lycopersicum*	22328/5.77	1	11
33	Superoxide dismutase [Cu-Zn] 1	P14830	*Solanum lycopersicum*	15408/5.83	4	38
50	PR10 protein	K4CWC4	*Solanum lycopersicum*	17472/5.44	8	54
66	Thioredoxin peroxidase 1	Q7Y240	*Solanum lycopersicum*	17540/5.18	7	45
152	Thiamine thiazole synthase, chloroplastic	K4CH99	*Solanum lycopersicum*	37677/5.42	11	37
175	Dehydrin	V5QQM9	*Solanum lycopersicum*	23138/5.17	8	40
176	Dehydrin	V5QQM9	*Solanum lycopersicum*	23138/5.18	6	25
199	Monodehydroascorbate reductase	Q43497	*Solanum lycopersicum*	47120/5.77	11	30
201	Monodehydroascorbate reductase	Q43497	*Solanum lycopersicum*	47120/5.77	11	32
241	Suberization-associated anionic peroxidase 1	P15003	*Solanum lycopersicum*	39295/4.92	5	16
242	Suberization-associated anionic peroxidase 1	P15003	*Solanum lycopersicum*	39295/4.93	5	16
**32.07 Detoxification**						
113	Ascorbate peroxidase	Q52QQ4	*Solanum lycopersicum*	27590/5.86	5	32
115	Cytosolic ascorbate peroxidase 1	Q3I5C4	*Solanum lycopersicum*	27733/5.61	10	44
116	Cytosolic ascorbate peroxidase isoform 3 (Fragment)	Q09Y78	*Solanum lycopersicum*	11131/8.96	7	56
117	Cytosolic ascorbate peroxidase 1	Q3I5C4	*Solanum lycopersicum*	27733/5.61	11	50
118	Cytosolic ascorbate peroxidase 1	Q3I5C4	*Solanum lycopersicum*	27733/5.61	6	34
120	LOW QUALITY PROTEIN: L-ascorbate peroxidase S, chloroplastic/mitochondrial-like	gi|460392865	*Solanum lycopersicum*	42710/8.12	14	36
129	Cytosolic ascorbate peroxidase 1	Q3I5C4	*Solanum lycopersicum*	27733/5.61	10	48

Table S1. Continued.

Spot no[z]	Matched protein	ID (NCBI)	Organism	Mr/pI	MP[y]	SC[z]
**34 Interaction with the cellular environment 34.11 Cellularsensingandresponse**						
10	Temperature-induced lipocalin	Q38JD4	*Solanum lycopersicum*	21509/6.16	10	51
61	Temperature-induced lipocalin	Q38JE1	*Solanum lycopersicum*	21303/5.96	5	31
65	Temperature-induced lipocalin	Q38JE1	*Solanum lycopersicum*	21303/5.96	7	43
**36 Interaction with the environment 36.20 Plant / fungal specific systemic sensing and response**						
133	Salicylate O-methyltransferase-like	gi|460373240	*Solanum lycopersicum*	42625/5.50	5	9
**41 Development 41.03 Plant development**						
105	Fruit protein pKIWI502-like	gi|460386364	*Solanum lycopersicum*	32756/7.10	10	39
155	Ripening regulated protein DDTFR10	Q9FR30	*Solanum lycopersicum*	22180/4.72	6	21
42 Biogenesis of cellular components 42.01 Cell wall						
167	UDP-glucose:protein transglucosylase-like protein SlUPTG1	Q6IV07	*Solanum lycopersicum*	41736/5.85	3	6
**42.03 Cytoplasm**						
72	**Translationally-controlled tumor protein homolog**	Q6DUX3	*Solanum lycopersicum*	19011/4.55	2	10
76	Translationally-controlled tumor protein homolog	Q6DUX3	*Solanum lycopersicum*	19011/4.55	1	5
**42.04 Cytoskeleton**						
143	Annexin	K4BSR4	*Solanum lycopersicum*	36291/5.55	22	62
171	Annexin	O81536	*Solanum lycopersicum*	35894/5.39	18	49
172	Annexin	O81536	*Solanum lycopersicum*	35894/5.39	10	29
203	Actin-7-like	gi|460408874	*Solanum lycopersicum*	41990/5.31	10	41
205	Actin-7-like	gi|460378623	*Solanum lycopersicum*	41963/5.31	16	55
206	Actin-7-like	gi|460408874	*Solanum lycopersicum*	41990/5.31	13	45
207	Actin-7-like	gi|460378623	*Solanum lycopersicum*	41963/5.31	18	57
210	Actin-7-like	gi|460378623	*Solanum lycopersicum*	41963/5.31	17	57
211	Actin-7-like	gi|460378623	*Solanum lycopersicum*	41963/5.31	14	49
**70 Subcellular location 70.27 Extracellular /secretion proteins**						
270	Ethylene overproducer-like 1	Q4F8Q5	*Solanum lycopersicum*	102181/5.57	11	12

Table S1. Continued.

Spot no[z]	Matched protein	ID (NCBI)	Organism	Mr/pI	MP[y]	SC[z]
**98 Un classified**						
3	Uncharacterized protein	gi|460387665	*Solanum lycopersicum*	205471/4.76	19	9
5	Uncharacterized protein	K4BDL5	*Solanum lycopersicum*	13679/5.85	2	11
6	Uncharacterized protein	K4B7E7	*Solanum lycopersicum*	13624/5.80	3	45
7	Uncharacterized protein	K4BRR1	*Solanum lycopersicum*	12502/6.75	5	52
11	Dihydroneopterin aldolase	Q6TFM2	*Solanum lycopersicum*	14639/6.29	5	44
15	Uncharacterized protein	K4BIC8	*Solanum lycopersicum*	16917/4.90	8	43
17	Uncharacterized protein	K4CND0	*Solanum lycopersicum*	10507/9.98	4	46
20	Predicted protein	gi|162667764	*Physcomitrella patens*	33133/9.50	10	23
22	Uncharacterized protein	K4D318	*Solanum lycopersicum*	14399/5.50	7	58
26	Uncharacterized protein	K4B3H9	*Solanum lycopersicum*	17390/5.58	6	49
28	Uncharacterized protein	K4CL22	*Solanum lycopersicum*	17381/6.75	4	42
31	Uncharacterized protein	K4BSP6	*Solanum lycopersicum*	21233/5.79	4	38
34	Uncharacterized protein	K4B3H9	*Solanum lycopersicum*	17390/5.58	12	58
35	Uncharacterized protein	K4BIL3	*Solanum lycopersicum*	19905/6.29	5	21
41	Uncharacterized protein	K4B3H9	*Solanum lycopersicum*	17390/5.58	8	46
42	Uncharacterized protein	K4D3F2	*Solanum lycopersicum*	24649/8.60	10	50
43	Uncharacterized protein	K4CF87	*Solanum lycopersicum*	55895/5.65	6	15
45	Uncharacterized protein	K4D399	*Solanum lycopersicum*	16706/6.20	9	69
46	Uncharacterized protein	gi|460410122	*Solanum lycopersicum*	123563/7.37	3	2
47	Predicted protein	gi|162667764	*Physcomitrella patens*	33133/9.50	5	13
54	Uncharacterized protein	K4B0D2	*Solanum lycopersicum*	19665/4.81	4	34
55	Uncharacterized protein	K4B1M2	*Solanum lycopersicum*	21631/7.90	12	51
56	Uncharacterized protein	K4BKP5	*Solanum lycopersicum*	21524/6.93	11	52
57	Uncharacterized protein	K4B2H1	*Solanum lycopersicum*	69241/8.44	12	14
62	Uncharacterized protein	K4B276	*Solanum lycopersicum*	25029/9.03	8	32

Table S1. Continued.

Spot no[z]	Matched protein	ID (NCBI)	Organism	Mr/pI	MP[y]	SC[z]
86	Uncharacterized protein	K4CXJ6	*Solanum lycopersicum*	21655/5.74	5	39
88	Uncharacterized protein	gi|460406712	*Solanum lycopersicum*	23448/8.80	8	43
91	Uncharacterized protein	K4BUN4	*Solanum lycopersicum*	28997/6.86	9	35
93	Uncharacterized protein	K4DC13	*Solanum lycopersicum*	22403/5.32	12	65
94	Uncharacterized protein	K4C3T2	*Solanum lycopersicum*	23708/5.98	8	62
109	Uncharacterized protein LOC101254447 isoform 2	gi|460375040	*Solanum lycopersicum*	37582/4.90	9	16
110	LePCL1 protein (Fragment)	Q4H2B2	*Solanum lycopersicum*	20959/4.90	4	11
119	Uncharacterized protein At2g37660, chloroplastic-like	gi|460401841	*Solanum lycopersicum*	33614/9.18	12	43
121	Uncharacterized protein At2g37660, chloroplastic-like	gi|460401841	*Solanum lycopersicum*	33614/9.18	11	35
126	Protein usf-like	gi|460389005	*Solanum lycopersicum*	31269/6.66	9	32
127	Protein usf-like	gi|460389005	*Solanum lycopersicum*	31269/6.66	9	32
130	Uncharacterized protein	gi|460413472	*Solanum lycopersicum*	82147/6.58	11	10
131	LePCL1 protein (Fragment)	Q4H2B2	*Solanum lycopersicum*	20959/4.90	4	11
132	Uncharacterized protein	K4B0D2	*Solanum lycopersicum*	98193/8.56	17	14
134	Uncharacterized protein	K4BV04	*Solanum lycopersicum*	34744/6.17	9	18
147	Uncharacterized protein	gi|460391932	*Solanum lycopersicum*	39177/9.93	10	26
156	Uncharacterized protein	gi|460398472	*Solanum lycopersicum*	35095/4.66	7	26
173	Uncharacterized protein	gi|460413263	*Solanum lycopersicum*	52779/9.59	5	8
179	Uncharacterized oxidoreductase At1g06690, chloroplastic-like	gi|460396131	*Solanum lycopersicum*	40913/7.57	9	24
181	Uncharacterized protein LOC101259630 isoform 2	gi|460385481	*Solanum lycopersicum*	32725/9.89	12	29
182	Uncharacterized protein LOC101259630 isoform 2	gi|460385481	*Solanum lycopersicum*	32725/9389	9	25
215	Uncharacterized protein LOC101259630 isoform 2	gi|460385481	*Solanum lycopersicum*	32725/9.89	10	20
216	Uncharacterized protein LOC101259630 isoform 2	gi|460385481	*Solanum lycopersicum*	32725/9.89	10	20
238	Uncharacterized protein LOC101259630 isoform 2	gi|460385481	*Solanum lycopersicum*	32725/9.89	10	27

Table S1. Continued.

Spot no[z]	Matched protein	ID (NCBI)	Organism	Mr/pI	MP[y]	SC[z]
239	Uncharacterized protein At5g39570-like isoform 2	gi|460372789	*Solanum lycopersicum*	39883/4.67	21	40
240	LePCL1 protein (Fragment)	Q4H2B2	*Solanum lycopersicum*	20959/4.90	5	14
256	Uncharacterized protein	gi|460373077	*Solanum lycopersicum*	25030/9.67	5	18
257	Uncharacterized protein LOC101259630 isoform 2	gi|460385481	*Solanum lycopersicum*	32725/9.89	12	27

[z]Spot numbers identified from 2D gel presented in Figure 2.

[y]Ratio of matched peptides that protein classified or unclassified on the function category was based on the 'GO-MIPS funcat conversion table' set up at the Munich Information Center for Protein Sequences (MIPS Institute).

[x]Sequence coverage (%).
